# Congenital Stationary Night Blindness: Clinical and Genetic Features

**DOI:** 10.3390/ijms232314965

**Published:** 2022-11-29

**Authors:** Angela H. Kim, Pei-Kang Liu, Yin-Hsi Chang, Eugene Yu-Chuan Kang, Hung-Hsuan Wang, Nelson Chen, Yun-Ju Tseng, Go Hun Seo, Hane Lee, Laura Liu, An-Ning Chao, Kuan-Jen Chen, Yih-Shiou Hwang, Wei-Chi Wu, Chi-Chun Lai, Stephen H. Tsang, Meng-Chang Hsiao, Nan-Kai Wang

**Affiliations:** 1 Department of Ophthalmology, Edward S. Harkness Eye Institute, Columbia University Medical Center, New York, NY 10032, USA; 2 Downstate Medical Center, College of Medicine, State University of New York, Brooklyn, NY 11203, USA; 3 Department of Ophthalmology, Kaohsiung Medical University Hospital, Kaohsiung Medical University, Kaohsiung 80756, Taiwan; 4 School of Medicine, College of Medicine, Kaohsiung Medical University, Kaohsiung 80708, Taiwan; 5 Institute of Biomedical Sciences, National Sun Yat-sen University, Kaohsiung 80424, Taiwan; 6 Department of Ophthalmology, Chang Gung Memorial Hospital, Linkou Medical Center, Taoyuan 33305, Taiwan; 7 College of Medicine, Chang Gung University, Taoyuan 33303, Taiwan; 8 Graduate Institute of Clinical Medical Sciences, College of Medicine, Chang Gung University, Taoyuan 33303, Taiwan; 9 Faculty of Health Sciences, Queen’s University, Kingston, ON K7L 3N6, Canada; 10 Division of Medical Genetics, 3billion, Inc., Seoul 06193, Korea; 11 School of Traditional Chinese Medicine, Chang Gung University, Taoyuan 33303, Taiwan; 12 Department of Ophthalmology, Chang Gung Memorial Hospital, Keelung 20401, Taiwan; 13 Department of Pathology and Cell Biology, Columbia University Medical Center, New York, NY 10032, USA

**Keywords:** inherited retinal disease, congenital stationary night blindness, retinitis pigmentosa

## Abstract

Congenital stationary night blindness (CSNB) is an inherited retinal disease (IRD) that causes night blindness in childhood with heterogeneous genetic, electrophysical, and clinical characteristics. The development of sequencing technologies and gene therapy have increased the ease and urgency of diagnosing IRDs. This study describes seven Taiwanese patients from six unrelated families examined at a tertiary referral center, diagnosed with CSNB, and confirmed by genetic testing. Complete ophthalmic exams included best corrected visual acuity, retinal imaging, and an electroretinogram. The effects of identified novel variants were predicted using clinical details, protein prediction tools, and conservation scores. One patient had an autosomal dominant CSNB with a *RHO* variant; five patients had complete CSNB with variants in *GRM6*, *TRPM1*, and *NYX*; and one patient had incomplete CSNB with variants in *CACNA1F*. The patients had Riggs and Schubert–Bornschein types of CSNB with autosomal dominant, autosomal recessive, and X-linked inheritance patterns. This is the first report of CSNB patients in Taiwan with confirmed genetic testing, providing novel perspectives on molecular etiology and genotype–phenotype correlation of CSNB. Particularly, variants in *TRPM1*, *NYX*, and *CACNA1F* in our patient cohort have not previously been described, although their clinical significance needs further study. Additional study is needed for the genotype–phenotype correlation of different mutations causing CSNB. In addition to genetic etiology, the future of gene therapy for CSNB patients is reviewed and discussed.

## 1. Introduction

Congenital stationary night blindness (CSNB) was first described by Florent Cunier in 1838 [1]. He wrote about a 23-year-old boy who suffered from night blindness; Cunier studied the boy’s family pedigree to identify 56 family members who experienced night blindness across 11 generations and traced their ancestry to Jean Nougaret. In the 20th century, Nettleship continued Cunier’s work and determined that the disease is inherited in a dominant manner and does not progress throughout one’s life [1]. Since then, the description of CSNB has evolved to include heterogeneous clinical presentations with distinct electrophysiological characteristics and multiple inheritance patterns.

There are four types of CSNB: Riggs, Schubert–Bornschein, fundus albipunctatus, and Oguchi disease [2]. Patients experience night blindness, myopia, strabismus, and/or nystagmus. The nystagmus is described as pendular, dysconjugate, oblique nystagmus with a high frequency and low amplitude [3]. These diseases can be distinguished based on the inheritance pattern, which can be autosomal dominant (AD), autosomal recessive (AR), or X-linked traits. On fundus exam, fundus albipunctatus has small white dots scattered across the posterior pole sparing the fovea while Oguchi disease has a gray-white metallic sheen that disappears after dark adaptation, a feature called the Mizuo–Nakamura phenomenon. Unlike these two diseases, Riggs and Schubert–Borstein have normal fundi and can be distinguished using full-field electroretinography (ff-ERG).

There are four components to an ff-ERG that measure the activities of different parts of the retina. In scotopic settings, a diffuse, full-field of light measures the activity of ON-bipolar cells, represented as a b-wave (Dark-adapted 0.01 ERG) [4]. With a stronger single flash, the combined activity of cones and rods are represented as the a-wave and ON- and OFF-bipolar cells as the b-wave (Dark-adapted 3.0 ERG) [4]. Riggs type has flat a-wave in dim flash and reduced a- and b-wave with a strong single flash [5]. In contrast, the Schubert–Bornschein type has normal a-wave and severely reduced b-wave, classically described as an electronegative waveform [6]. The other two measurements are measured after light adaptation with a strong single flash stimulus and 30 Hz flicker. While the Riggs type has a normal photopic response, the Schubert–Bornschein type has abnormal photopic findings. The two subgroups of the Schubert–Bornschein type are further divided into two subtypes with distinctive ff-ERG findings: complete or incomplete. The complete subtype has abnormal ON-bipolar cells while the incomplete subtype has abnormal ON- and OFF-bipolar cells, and their ff-ERG findings differ accordingly [7]. After photopic adaptation, the complete subtype has a preserved a-wave and a widened trough with a sharply rising b-wave after a single flash (Light-adapted 3.0 ERG (single-flash cone response)) [7]. With 30 Hz, the complete subtype has a flattened trough with or without a mild implicit time shift. The incomplete subtype has a reduced a- and b-wave such that the b:a ratio is markedly reduced and a 30 Hz ERG with a reduced amplitude and distinctive bifid waveforms (Light-adapted 3.0 ERG (30 Hz flicker)) [7]. The ff-ERG findings of CSNB are summarized in Table 1.

In addition to ff-ERG findings, the subtypes of CSNB can be distinguished by different inheritance patterns: the Riggs type can be inherited in an AD or AR manner while the Schubert–Bornschein type CSNB can be inherited in an AR or X-linked manner (Table 1) [8].

This study describes a case series of seven Taiwanese CSNB patients, including their clinical presentation, diagnostic imaging findings, electrophysical findings, and genetic variants. This is the first cohort study of genetically confirmed CSNB patients from Taiwan.

## 2. Results

### 2.1. Demographics and Genetic Results

Patient demographics are summarized in Table 2. All patients were male with a mean age of 17.9 years. Patients’ ages ranged from 7 to 28, with a median age of 22. Patient 1 has a heterozygous variant in the *RHO* gene, which causes AD CSNB. Patient 2 carries two variants in the *GRM6* gene, which causes AR complete CSNB (cCSNB). The two variants, 28 bp apart from each other and located in exon 3, were determined to be *in trans* by TA-cloning. Patients 3 and 4 are siblings with variants in the *TRPM1* gene, which also causes AR cCSNB. The two variants were determined to be *in trans*. Patient 5 is from another family, who also carries two variants in the *TRPM1* gene. One variant is inherited from the mother and the other variant, which is not inherited from the mother, could be de novo or inherited from the father. Patient 6 carries a heterozygous variant in the *CACNA1F* gene, which causes X-linked incomplete CSNB (iCSNB). Patient 7 carries a hemizygous variant in the *NYX* gene, which causes X-linked cCSNB. The genetic test results are summarized in Table 2.

### 2.2. Clinical Features

**Figure 1:**
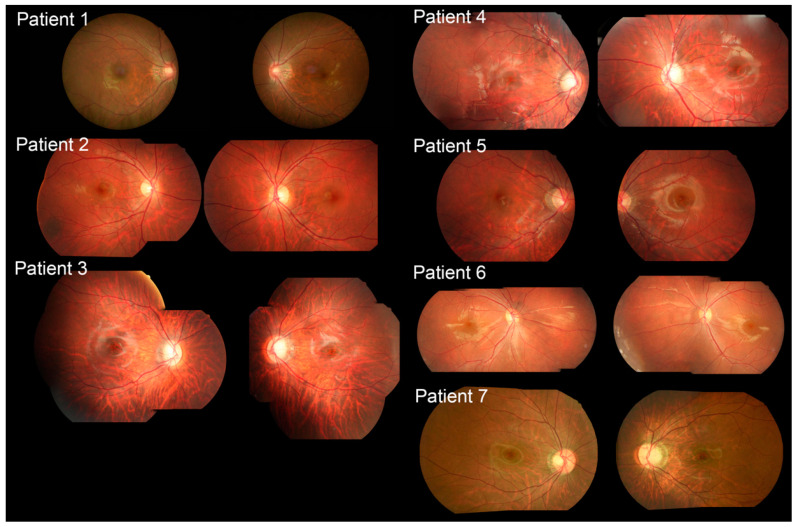
Color fundus imaging. Patient 1 presented with normal fundus photography without any bone spicules, arteriolar attenuation, or disc pallor. Color fundus imaging showed peripapillary atrophy and a temporal crescent in both eyes. Patient 2 had normal color fundus photography except a nevus in the right periphery. Patient 3′s color fundus photography showed tessellated fundus and a large optic nerve in both eyes with slightly enlarged cupping in the left eye. Patients 4–7 presented with normal fundus photography.

**Figure 2:**
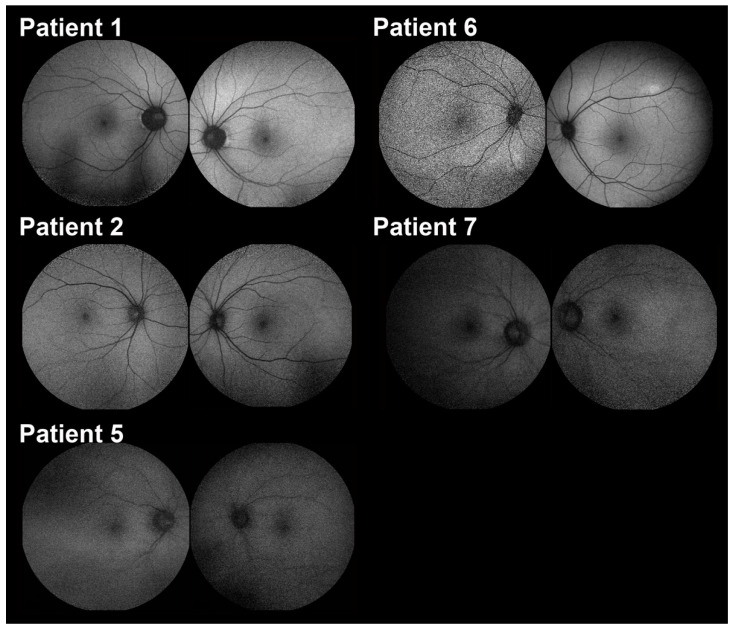
Short-wave autofluorescence (SW-AF) imaging. Each number corresponds to the patient number, with the right eye first followed by the left eye. Patients 1, 2, 6, and 7 showed normal findings. The image for patient 5 is qualitatively within the normal for AF. From the available images, patients 5, 7, and the right eye of patient 6 could be classified as granular; however, this is most likely artefactual.

**Figure 3:**
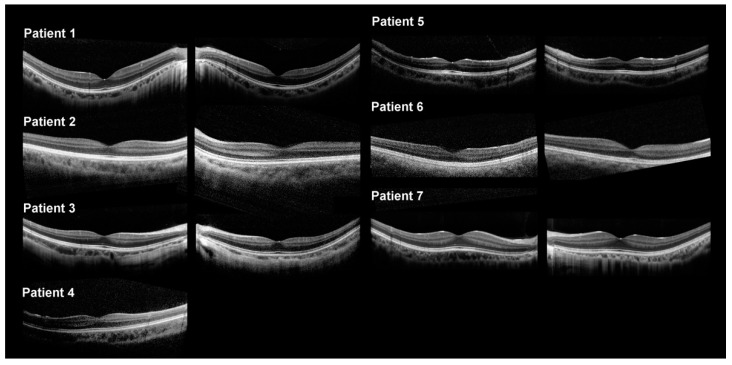
Spectral domain optical coherence tomography (SD-OCT) imaging. Each number corresponds to the patient number, with the right eye first followed by the left eye. Patient 1 had an intact ellipsoid (EZ) line and thin choroid in both eyes. Patient 2 had intact inner and outer segment lines. Patient 3 had intact EZ lines and a normal retinal architecture in both eyes. Patient 4 had an intact EZ line in the right eye, and SD-OCT in the left eye showed no obvious defect in the anatomical structure except for a thinner retina. Patient 5 had a normal SD-OCT. Patient 6 had an overall thin retina. Patient 7 had dome-shaped macula in his right eye, and an intact EZ line in both his eyes.

**Figure 4:**
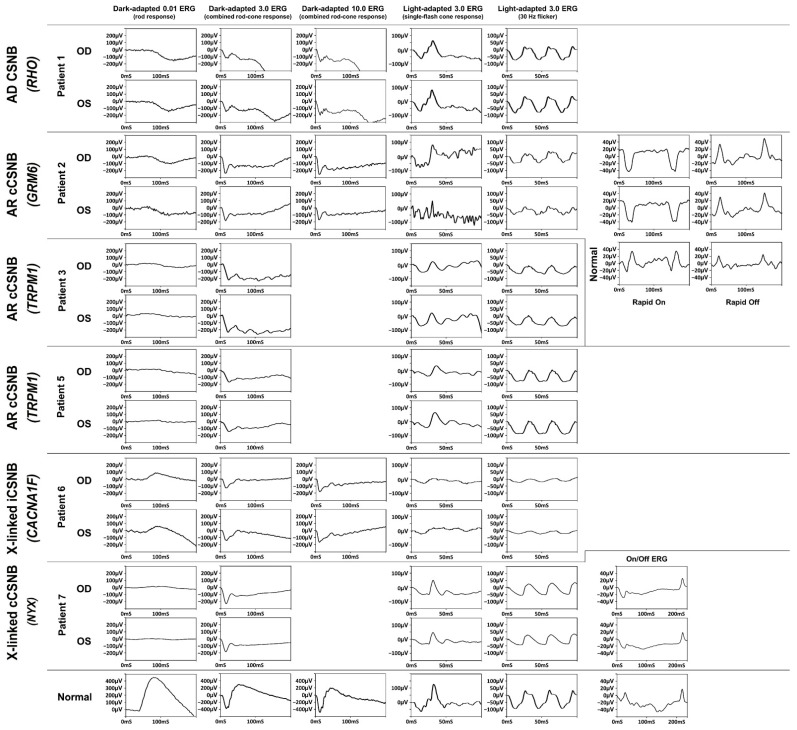
Patients’ full field electroretinogram waveforms.

The best corrected visual acuity (BCVA) ranges from 20/20 to 20/500. Patient 1 had AD CSNB with the *RHO* variant. The patient presented with a BCVA of 20/20 in both eyes with a long history of nyctalopia since elementary school and no amblyopia. The patient had diagnostic imaging findings within normal limits (Figure 1, Figure 2 and Figure 3). Patient 1 had extinguished dark-adapted (DA) 0.01 ERG, electronegative DA 3.0 ERG, and 10.0 ERG. The photopic response was normal (Table 3, Figure 4).

Patients 2–5 and 7 were diagnosed with cCSNB due to variants in *GRM6*, *TRPM1*, and *NYX*. Patient 2 presented with BCVA of 20/200 bilaterally with a history of poor vision since childhood. Diagnostic imaging, including spectral domain optical coherence tomography (SD-OCT), of patients 1, 3, 4, 5, and 7 showed decreased choroidal thickness (Figure 1, Figure 2 and Figure 3). Patient 2 had extinguished DA 0.01 ERG and electronegative DA 3.0 ERG. Photopic responses are in the normal range (flash cone 60–120 µV, 30 Hz: 40–160 µV), although LA 3.0 ERG of the left eye could not be analyzed. His abnormal rapid on responses with normal rapid off responses indicate only ON-bipolar cell dysfunction. Patient 3 was presented by the parents for abnormal gazing and poor night vision and mild myopia. He was examined with his younger brother, patient 4, who had nystagmus and decreased night vision. Patient 3 had BCVA of 20/30 in the right eye and 20/100 in the left eye. Patient 3 had extinguished DA 0.01 ERG, electronegative DA 3.0 ERG, and reduced photopic ERG. DA 10.0 ERG was not performed for patients 2 and 3 at this time, as it was before 10.0 ERG was included in the International Society for Clinical Electrophysiology of Vision (ISCEV) protocol. Patient 4 had BCVA of 20/200 in both eyes, but he had amblyopia corrected with glasses. Patient 4 was too young to undergo ff-ERG. Patient 5 presented with progression of blurry vision for one year with pre-existing nyctalopia and nystagmus since childhood. His BCVA was 20/200 in both eyes. No consanguinity was reported in the family, and no one else had similar visual problems. Patient 7 presented with a family history of nystagmus and BCVA of 20/20 bilaterally. The patient has experienced decreased night vision since childhood and was originally diagnosed with retinitis pigmentosa (RP) despite no abnormal retinal findings. No consanguinity was reported in the patients’ families. Among those with cCSNB, patients 2, 3, 5, and 7 had a reduced b-wave amplitude in DA 0.01, 3.0, and 10.0 ERG. They also had electronegative responses in 3.0 ERG and 10.0 ERG, broadened a-wave, and rapidly rising b-wave in light-adapted (LA) 3.0 ERG. The amplitudes of the b-waves in the LA 30 Hz flicker were in the normal range.

Diagnostic imaging, including color fundus, was not remarkable other than in patient 2, who had a nevus in the periphery (Figure 1). Short-wave autofluorescence (SW-AF) did not reveal focal areas of increased or decreased autofluorescence signal in any patients, although some were granular likely due to artefacts. Patients 5 and 7 demonstrated relatively reduced background autofluorescence (Figure 2). SD-OCT of all patients showed a normal architecture without any disruption to the ellipsoid zone or any hyperreflective deposits (Figure 3). Patient 4 is missing an SD-OCT image of the left eye.

Patient 6 with a medical history of congenital atrial septal defect and ventricular septal defect presented to the clinic with a BCVA of 20/500 in both eyes. The patient was diagnosed with iCSNB with a variant in *CACNA1F*. He had congenital nystagmus, hypermetropia with esotropia, and color vision deficiency. Patient 6 had a reduced b-wave in DA 0.01 ERG, reduced a- and b-wave amplitudes, and electronegative ERG (b < a) was more obvious in DA 10.0 ERG. The LA 3.0 ERG and 30 Hz showed a reduced amplitude of b-waves.

## 3. Discussion

### 3.1. Pathophysiology and Clinical Presentations

The seven patients diagnosed with CSNB at Chang Gung Memorial Hospital (CGMH) Linkou, the biggest tertiary referral hospital in Taiwan, which sees 4 million patients per year, highlights the rarity of this disease. CSNB is a clinically and genetically heterogeneous disease that has had various presentations in diverse populations with different variants. The seven Taiwanese patients with CSNB in this study share similar phenotypic characteristics as previously described in other populations, with some variable features that may shed some light on genotype–phenotype correlations (Table 2, Table 4 and Table 5).

Patient 1 carries a variant in the *RHO* gene, which is the most commonly mutated gene for autosomal dominant RP (ADRP). The *RHO* gene encodes for rhodopsin protein, which is a G-protein receptor that plays a critical role in rod phototransduction. Pathogenic *RHO* variants could cause CSNB and RP. Although both can be inherited as the AD pattern, they have different features in ff-ERG and clinical courses. Unlike patients with RP, patients with *RHO*-related CSNB experience nyctalopia that does not progress, as seen in patient 1. The ff-ERG findings of patient 1 showed extinguished DA 0.01 ERG and a reduced a-wave and b-wave in DA 3.0 ERG and 10.0 ERG, which reflects rod dysfunction in Riggs-type ERG. Unlike *RHO*-related ADRP, the LA ERG of patient 1 was normal, indicating that the cone responses in *RHO*-related CSNB are unaffected.

Previous descriptions of AD CSNB revealed no association with myopia, nystagmus, and amblyopia unlike other types of CSNB [9,10,11,12]. Similar to the Slovenian cohort with AD CSNB caused by *RHO* variants with 20/20 visual acuity, patient 1 had good visual acuity (Table 5) [9]. Patient 1′s myopia of −3.75 and −3.25 diopters was in the range of average myopia in Taiwan and does not seem related to the diagnosis of AD CSNB (Table 5) [13]. For clinical genetic testing, physicians can narrow down the testing gene to *RHO* in patients with an autosomal dominant family history, electronegative ERG with a normal cone system function, and lack of amblyopia. [8]. In the literature, four *RHO* missense variants have been reported in AD CSNB, and all these variants are located in the transmembrane domain [14]. Although it remains unknown why these specific *RHO* variants cause characteristic phenotypes in CSNB that are different from the phenotypes in ADRP, further studies are needed to elucidate the binding site of RHO protein with GRK1, SAG, and GNAT1.

Nystagmus is another clinical feature associated with CSNB that prompted pediatric patients in our cohort to seek ophthalmic care. Nystagmus is classified based on its onset: infantile nystagmus appears in the first 3 to 6 months after birth while acquired nystagmus appears later. The former has been associated with congenital causes of low vision in early life, such as albinism, optic nerve hypoplasia, cataracts, or CSNB. The acquired disease decreases vision due to rapid oscillation of the image across the fovea [15]. In our patient cohort, patients 1 and 7 were the only patients without nystagmus with mean BCVA of 20/20 compared to patients 2–6, who had nystagmus with BCVA ranging from 20/200 to 20/500.

*GRM6*, *TRPM1*, and *NYX* are genes affected in our cCSNB patients that are involved in glutamate signaling between bipolar cells and photoreceptors. *GRM6* encodes for glutamate receptors on bipolar cells and is inherited in an AR pattern [16]. *TRPM1* encodes for an ion-conducting membrane channel that is responsible for depolarizing ON-bipolar cells to light stimuli [17], and *NYX* encodes for nyctalopin protein, which is responsible for the correct position of the TRPM1 channel at synapses [18]. The patients present with cCSNB features and with an X-linked or AR inheritance pattern. On ff-ERG, patients have no b-wave in DA 0.01 and have electronegative ERG with normal a-wave and attenuated b-wave in DA 3.0 or 10.0, as can be seen in patients 2, 3, 5, and 7. The amplitudes of LA 3.0 and 30 Hz are usually within normal limits. The ff-ERG findings of cCSNB patients with *GRM6*, *TRPM1*, and *NYX* were in accordance with previously published findings of cCSNB (Figure 4, Table 1).

Unlike the ff-ERG findings, the clinical features of this patient cohort were variable compared to previously described patient cohorts (Table 5). While patients 2′s vision was relatively worse than the mean BCVA with the same mutations in the literature, the patient’s visual acuity was within the range of BCVA in CSNB patients with the same mutations [20]. Patients 2–5 had BCVA worse than 20/125 and patient 7 had BCVA of 20/20 bilaterally [22]. Patient 2 with the *GRM6* variant had amblyopia and nystagmus similar to the patient cohorts previously published but poorer visual acuity than previously reported [20]. Patients with the *TRPM1* variants (patients 3–5) in this cohort have a similar visual acuity found in European and Korean cohorts [19,23,24]. In a Dutch population, those with cCSNB had a mean visual acuity of about 20/50 and mean refractive error of −3.75 D [10]. Furthermore, Patients 3–5 have myopia, nystagmus, and strabismus, which have been previously associated with CSNB due to the *TRPM1* mutation. Patient 7 with the *NYX* variant had better visual acuity than the Chinese patient cohort [25]. All patients in our cohort except patients 1 and 7 had amblyopia (Table 5). The sample size is too small to make conclusions, and further study is needed to determine the genotype–phenotype correlations.

The *CACNA1F* gene, mutated in patient 6, encodes a calcium voltage-gated channel subunit, which mediates neurotransmitter release in scotopic conditions. The mutation is inherited in an X-linked recessive manner and affects both ON- and OFF- bipolar cells. The ff-ERG has a normal a-wave and reduced b-wave in DA 3.0 and 10.0 and reduced LA 3.0 and LA 30 Hz whose shape is commonly bifid. The clinical features associated with X-linked iCSNB in the Dutch population were average visual acuity of 20/55 and myopia of −5.56 D, although 22% of the population presented with hyperopia (Table 5) [10]. Other reported symptoms in this population were photophobia and color blindness. In a Korean cohort with iCSNB, the average visual acuity was 20/123 and refractive error was −7 [19]. While patient 6 had similar color blindness as seen in the Dutch and Korean patients with CSNB, he presented with poorer visual acuity, hyperopia, strabismus, and nystagmus [10]. It is worthy to note that patient 6 has esotropia and hyperopia, which could be another reason for poor BCVA. He had variant c.2231C>A p.Ala744Asp, which causes amino acid change from a non-polar amino acid (Ala) to a charged amino acid (Asp) at a position that is highly conserved (phyloP100way = 9.68 is greater than 7.2). This variant has not been published previously.

In summary, our patient cohort with variants in *GRM6*, *TRPM1*, and *CACNA1F* had BCVA worse than those reported previously while those with *RHO* and *NYX* matched those previously (Table 5). As for refractive error, most of the patients with CSNB reported in the literature were myopic, and patients with the *NYX* variant were more myopic than those with other variants (Table 5) [21]. Those with the *GRM6*, *TRPM1*, or *CACNA1F* variants had both amblyopia and nystagmus (Table 5). While the sample size is too small to make conclusions, this patient cohort from Taiwan will be valuable when studying genotype–phenotype correlations in the future. Despite the catchment area of 4 million patients per year at CGMH Linkou Medical Center, only 7 patients have been diagnosed with CSNB based on ff-ERG, highlighting the lack of adequate diagnostics in primary care. The generally benign diagnostic images and availability of ff-ERG may be factors in the infrequency of diagnoses. Therefore, ff-ERG remains an important exam to diagnose CSNB.

### 3.2. Variant Interpretation and Clinical Significance Evaluation

Patient 1 carries a heterozygous variant *RHO* c.281C>T (p.Thr94Ile), which has been reported as pathogenic by ClinVar (Variation ID: 13054) [12]. This variant has been proposed to be associated with a constitutive activation of transducin by the altered rhodopsin protein [26].

**Figure 5:**
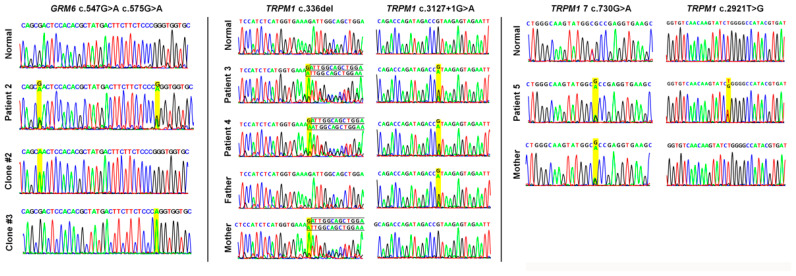
Phase determination for compound heterozygous patients 2–5.

Patient 2 carries two variants in the *GRM6* exon 3, c.547G>A (p.Asp183Asn) and c.575G>A (p.Arg192Gln), both have been reported as VUS in ClinVar (Variation ID: 1428176 and 1443702). These two variants are *in trans* compound heterozygous according to the TA cloning result (Figure 5). These two variants are not commonly found in the general population, suggesting that they are not common benign variants. Additionally, they are predicted to be deleterious by multiple prediction tools (Table 4). Although these two variants have not been functionally characterized, they likely alter the functional domain and further contribute to the phenotype. Patients 3 and 4 are siblings, carrying identical *TRPM1* compound heterozygous variants c.336del (p.Asp113lleTer10) and c.3127+1G>A *in trans*, confirmed by Sanger sequencing of their parents (Figure 5). These two variants have been reported as pathogenic and likely pathogenic in ClinVar (variation ID: 1431480 and 1517049). The c.3127+1G>A variant is located in the canonical splice site, which is expected to result in exon skipping. The truncating variant c.336del (p.Asp113IlefsTer10) is expected to result in mRNA decay. Both variants are expected to be disease-causing by loss of function. Patient 5 carries two *TRPM1* variants c.2921T>G (p.Leu974Arg) and c.730G>A (p.Ala244Thr). The c.730G>A (p.Ala244Thr) variant has been reported as VUS in ClinVar (variation ID: 934531). While the variant c.730G>A (p.Ala244Thr) is maternally inherited (Figure 5), whether the variant c.2921T>G (p.Leu974Arg) is de novo or paternally inherited is unknown as the paternal DNA was unavailable. Although these two variants are predicted to be tolerated by some prediction tools and have not been functionally characterized, they are not commonly found in the general population, suggesting they are less likely to be tolerated (Table 4). Therefore, these two variants are likely disease-causing by altering the functional domain. Patient 6 carries a hemizygous variant *CACNA1F* c.2231C>A (p.Ala744Asp), inherited from his unaffected mother (confirmed by Sanger sequencing). However, this patient’s granduncle was also diagnosed with night blindness, strongly indicating that this variant is disease-causing by an X-linked pattern. Patient 7 carries a hemizygous truncating variant in the *NYX* gene, c.217_218insA(p.Gly73GlufsTer42), which is expected to result in nonsense-mediated mRNA decay. Therefore, this variant is likely disease-causing by loss of function.

### 3.3. Clinical Therapeutics

With the recent advances in gene therapy for the biallelic *RPE65* mutation, finding therapeutics for other inherited retinal diseases (IRDs) is now a possibility. Several approaches of gene therapies for IRDs exist, using Adeno-associated viral vectors (AAVs) or Lentivirus-mediated gene augmentation (providing normal cDNA, ex *RPE65*), and gene editing (CRISPR to knockout point mutation in deep intronic regions, e.g., *CEP290* causing LCA110). Although there are currently no clinical human trials for CSNB, previous work in mouse models has shown that gene therapy delivered with viral vectors can restore visual function [27,28]. Unlike other IRDs, CSNB has not received much therapeutic effort due to its clinical course. Most of the CSNB patients develop poor vision in infancy that leads to nystagmus before the age of two and subsequently develop amblyopia. Amblyopia occurs when the brain cannot receive clear images from the eyes, and the visual cortex does not develop in conjunction with visual stimuli [29]. The reversal of amblyopia and nystagmus depends not only on retinal function but also the brain maturity, which is achieved around seven years of age [30]. Achromatopsia is another disease called stationary day blindness. Similar to patients with CSNB, patients with achromatopsia have nystagmus and amblyopia and had some positive outcomes in gene augmentation clinical trials. A phase I/II gene therapy trial in 2020 by Fischer et al. and in 2021 by Reichel et al. targeting achromatopsia due to *CNGA3* demonstrated improved cone-mediated vision [31,32]. Both trials suggested that amblyopia may be a limiting factor in truly assessing the benefit of gene augmentation and suggested further research assessing the effect of gene augmentation in younger patients during the optimal therapeutic window for amblyopia [31,32].

In comparison to those with achromatopsia or CSNB, RP patients are not as limited in the therapeutic window because RP patients initially have unaffected vision, with or without nyctalopia initially, that eventually progresses to visual field constriction and central visual loss. As a result, clinical trials whose purposes are to prevent progression of RP and restore visual function may not be as applicable to a non-progressive retinal disease with amblyopia. However, several reports suggested that CSNB may not be as stationary as previously described. Reports have described deterioration of visual function and retinal findings in CSNB patients with *GRM6* and *CACNA1F* mutations whose visual acuity, retina, and optic nerve deteriorated over time [33,34]. While the exact genotype–phenotype mechanism of progressive CSNB needs further investigation, these cases emphasize the need for therapeutic efforts in CSNB. In addition, the randomized Pediatric Eye Disease Investigator Group (PEDIG) clinical trial for amblyopia showed that visual function may even be improved in patients 13–17 years old, with improvement of vision greater than one line [35]. The Amblyopia Preferred Practice Pattern guideline published by the American Academy of Pediatric Ophthalmology in 2018 suggested treatment of amblyopia up to 10 years of age [36]. The youngest patient injected for the Luxturna clinical trial was 8 years old [37]. With a more streamlined subretinal surgical protocol, gene therapy for CSNB and achromatopsia patients earlier in life may be a possibility [38]. With the forementioned developments, the clinical therapeutic target, and the need for CSNB patients should be re-evaluated.

Pre-clinical studies of the efficiency of gene therapy in animal models have been performed not only to gain a better understanding of disease mechanisms but also to assess the therapeutic potential. Since 2015, various groups have tested the efficacy of AAV-mediated gene augmentation CSNB mouse and dog models with *NYX*, *LRIT3*, and *GRM6* knockouts [27,28,39,40,41,42]. All found structural recovery, although the ff-ERG response recovery had a wide spectrum of response [27,28,39,40,41,42]. In addition to gene therapy, there has been significant work on non-gene specific therapeutic options. In 2012, Pearson et al. showed a successful improvement of rod-mediated vision in the *Gnat1*^−/−^ mouse after rod photoreceptor transplant, which was not only able to form second-order synapses with bipolar and horizontal cells but was also able to show neural activity in the V1 cortical area [43]. The treated mouse had a dim-flash optokinetic response similar to the wildtype mouse’s and had improved visual task of navigating through the maze compared to the untreated mouse [43]. While the therapeutic methods are currently still in the early phase, a greater understanding of pathophysiology has been achieved with naturally occurring and designed animal models. The preclinical study results are optimistic and while further studies on the optimization and therapeutic window are wanted, clinical application of gene therapy for CSNB may be on the horizon.

## 4. Methods and Materials 

### 4.1. Patients

A total of seven patients with a diagnosis of CSNB were recruited from CGMH, Linkou, based on clinical exam, family history, and genetic testing. A consent form approved by the institutional review board of CGMH, Linkou (protocol No. 201601569B0C602) was used to obtain informed consent. All procedures followed the tenets of Helsinki.

### 4.2. Ophthalmologic Examination

Ophthalmic examination involved measurement of BCVA, intraocular pressure, slit lamp, and fundus examination. Patients underwent SW-AF and SD-OCT as described previously and color fundus imaging (TRC-50EX; Topcon, or Nonmyd α-DIII; KOWA, Tokyo, Japan) [44]. The ff-ERG was performed using Burian Allen contact lens electrodes, according to the ISCEV standards using a Utas-E3000 system (LKC Technologies, Inc., Gaithersburg, MD, USA) at Taipei CGMH or the Diagnosys Espion system (Diagnosys LLC, Lowell, MA, USA) at CGMH, Linkou as previously described [45,46].

### 4.3. Genetic Analysis

Whole exome sequencing was performed using DNA extracted from the peripheral blood of the patients [47]. For compound heterozygous variants in the *GRM6* and *TRPM1* gene, phases were determined by Sanger sequencing of the variants from the parents or TA-cloning, a subcloning technique when parents’ DNA was not available. Patient 2 has compound heterozygous variants in the *GRM6* gene; TA-cloning was used to subclone the PCR products because the parents’ DNA was not available, and the two variants were both located in exon 3 of the *GRM6* gene with 28 bp apart. DNA substrate (330 bps) was amplified from genomic DNA of patient 5 using the primers: 5′–TACCCTCCCTCTCTTGAGTTACTGA–3′ (forward) and 5′–CCGGGCCCACACTATGTAGAC–3′ (reverse). An amplicon of 330 base pairs was gel purified by a QIAquick Gel Extraction kit (Qiagen, Cat. No. 28704, Hilden, Germany), cloned into pGEM^®^-T Easy Vector Systems (A1360, Promega, Madison, WI, USA), and sequenced with the primers: 5′–GTAAAACGACGGCCAGT–3′ (M13 forward) according to the manufacturer’s instructions. Patients 3 and 4 are siblings with compound heterozygous variants in the *TRPM1* gene. DNA was amplified from patients 3 and 4 and their parents using the primers: 5′–TCACTCCCCTTTGACACGAGAAG–3′ (forward) and 5′–GAGTTCTGGGTGGTACATTGATTATCG–3′ (reverse) for exon 5; primers: 5′–GCCAGGGTGAACTTGGCCCA–3′ (forward) and 5′– AAAATAATTTGATGCCTATGGTGGAGTGAC–3′ (reverse) for exon 23 of the *TRPM1* gene. For patient 5, who has compound heterozygous variants in the *TRPM1* gene, DNA was amplified from patient 5, and his mother using the primers: 5′– TGCTTGCTTCCCTTGTTGGTCTTA–3′ (forward) and 5′–TCCTTCTCAGCCTTGTTTCCACTG–3′ (reverse) for exon 7; primers: 5′–ACGGCATCTCAGTTTATTGTTCTTTGG–3′ (forward) and 5′–AACTACAGCCAAAGTCTCTCTGGAT–3′ (reverse) for exon 22 of the *TRPM1* gene.

### 4.4. Functional Impact Prediction of Missense Variants

All missense variants that were classified as “variants of uncertain significance (VUS)” according to the American College of Medical Genetics (ACMG) guidelines were assessed with ANNOVAR using the pathogenicity scores of SIFT [48], PolyPhen [49], CADD [50], REVEL [51], MetaLR [52], and MutationAssessor [48]. The pathogenic consequences are predicted for variants with scores <0.05 for SIFT, ≥0.5 for PolyPhen-2, >20 for CADDv1.6, >0.5 for Rare Exome Variant Ensemble Learner (REVEL), with higher scores between 0 and 1 indicating pathogenicity for MetaLR. The variants’ minor allele frequencies (MAFs) were derived from the gnomAD dataset (gnomad.broadinstitute.org; v2.1.1). The conservation scores were graded using phyloP100way [53].

## 5. Conclusions

This is the first report of patients with CSNB in Taiwan. These patients have variants in *RHO*, *GRM6*, *TRPM1*, *CACNA1F*, and *NYX*. Particularly, variants in *TRPM1*, *NYX*, and *CACNA1F* in our patient cohort have not been reported before, bringing new insights into genetic etiology and genotype–phenotype correlations. While the ff-ERG findings of these patients were in accordance with the previously described findings in Riggs and Schubert–Bornschein type CSNB, the visual acuity was notably variable. These patients illustrate the need for further investigation into the pathophysiology and provide much-needed data for genotype–phenotype assessment.

## Figures and Tables

**Table 1:** Summary of congenital stationary night blindness (CSNB) types, inheritance patterns, and electroretinogram findings.

Inheritance Pattern	Type	Genes	ERG Findings
Autosomal dominant	Riggs	*RHO* *GNAT1* *PDE6B*	DA 0.01: non-detectable DA 3.0 and 10.0: decreased with decreased b:a ratio (possibly electronegative) LA 30 Hz and 3.0: normal photopic ERG
Autosomal recessive	Abnormal fundus	*SAG* *GRK1* *RDH5* *RLBP1* *RPE65*	DA 0.01: reduced DA 3.0 and 10.0: decreased with decreased b:a ratio (possibly electronegative); recovery of scotopic ERG after prolonged dark adaptation LA 30 Hz and 3.0: normal photopic ERG
	Complete	*GRM6* *TRPM1* *GPR179* *LRIT3*	DA 0.01: absent DA 3.0 and 10.0: decreased with decreased b:a ratio (electronegative) LA 30 Hz: normal amplitude, possibly increased latency LA 3.0: normal a-wave with wide trough; sharply rising b-wave with no oscillatory potentials with decreased reduced b:a ratio
	Incomplete	*CABP4* *CACNA2D4*	DA 0.01: reduced amplitude DA 3.0: slightly decreased a-wave, decreased a-wave DA 10.0: normal a-wave bright flash, reduced b-wave (electronegative) bright flash scotopic LA 30 Hz: reduced with increased latency, bifid peaks LA 3.0: reduced amplitude, decreased b:a ratio
	Riggs	*SLC24A1* *GNAT1*	DA 0.01: non-detectable DA 10.0: decreased bright flash scotopic ERG with decreased b:a ratio (possibly electronegative) LA 30 Hz and 3.0: normal photopic ERG
X-linked	Complete	*NYX*	DA 0.01: absent DA 10.0: decreased with decreased b:a ratio (electronegative) LA 30 Hz: normal amplitude, possibly increased latency LA 3.0: normal a-wave with wide trough; sharply rising b-wave with no oscillatory potentials with decreased reduced b:a ratio
	Incomplete	*CACNA1F*	DA 0.01: reduced amplitude DA 10.0: normal a-wave bright flash, reduced b-wave (electronegative) bright flash scotopic LA 30 Hz: reduced with increased latency, bifid peaks LA 3.0: reduced amplitude, decreased b:a ratio

**Table 2:** Demographic summary of our patients diagnosed with congenital stationary night blindness (CSNB). The mean age at diagnosis was 17.9 years.

Patient No.	Age	Sex	BCVA OD OS	Refractive Error (D)	Clinical Diagnosis	Gene (OMIM No.)	Transcript	Variants	ACMG Classification	Zygosity
1	22	M	20/20 20/20	−3.75 −3.25	AD CSNB	*RHO* (180380)	NM_000539.3	c.281C>T (p.Thr94Ile)	Likely pathogenic	Heterozygous
2	28	M	20/200 20/200	−1.75 −1.25	AR cCSNB	*GRM6* (604096)	NM_000843.4	c.547G>A (p.Asp183Asn) c.575G>A (p.Arg192Gln)	VUS VUS	Compound Heterozygous
3 ^§^	7	M	20/30 20/100	−5.0 −5.0	AR cCSNB	*TRPM1* (603576)	NM_001252024.2	c.336del (p.Asp113IlefsTer10) c.3127+1G>A	Pathogenic Pathogenic	Compound Heterozygous
4 ^§^	5	M	20/200 20/200	−5.0 −5.25	AR cCSNB	*TRPM1* (603576)	NM_001252024.2	c.336del (p.Asp113IlefsTer10) c.3127+1G>A	Pathogenic Pathogenic	Compound Heterozygous
5	15	M	20/200 20/200	−8.5 −9.0	AR cCSNB	*TRPM1* (603576)	NM_001252024.2	c.2921T>G (p.Leu974Arg) c.730G>A (p.Ala244Thr)	VUS VUS	Unknown *
6	26	M	20/500 20/500	+4.0 +4.25	X-linked iCSNB	*CACNA1F* (300110)	NM_001256789.3	c.2231C>A (p.Ala744Asp)	Likely pathogenic	Hemizygous
7	22	M	20/20 20/20	−6.25 −8.25	X-linked cCSNB	*NYX* (300278)	NM_001378477.3	c.217_218insA (p.Gly73Glufs * 42)	Likely pathogenic	Hemizygous

BCVA = best corrected visual acuity; AD = autosomal dominant; AR = autosomal recessive; VUS = variants of uncertain significance; OD = right eye; OS = left eye; cCSNB = complete congenital stationary night blindness; iCSNB = incomplete congenital stationary night blindness; D = diopter; No. = number. ^§^ Patients 3 and 4 are siblings. * phase testing was not possible.

**Table 3:** Summary of the full field electroretinogram (ff-ERG) results of our patients diagnosed with congenital stationary night blindness (CSNB).

Patient No.	Dark-Adapted 0.01 ERG			Dark-Adapted 3.0 ERG		Light-Adapted ERG			
	a Wave (μV) OD/OS	b Wave (μV) OD/OS	IT (ms) OD/OS	a Wave (μV) OD/OS	b Wave (μV) OD/OS	a Wave(μV) OD/OS	b Wave (μV) OD/OS	30 Hz Flicker (μV) OD/OS	30 Hz Flicker IT (ms) OD/OS
Patient 1	−8.6/−12.7	4.1/3.7	80/81	−119.1/−144.7	40.5/48.7	−59.2/−68.6	130.7/153.6	94.2/118.8	27/27
Patient 2	−2.6/−1.3	33.35/19.36	92/90	−228.6/−180.1	120.0/106.4	−19.1/−33.4	83.2/70.0	71.9/56.8	30/28
Patient 3	−5.2/−7.2	13.3/24.7	64.5/64	−145.0/−211.7	81.8/96.9	−40.0/−47.6	72.3/88.0	54.2/60.8	31.5/31
Patient 4 *	NA	NA	NA	NA	NA	NA	NA	NA	NA
Patient 5	−2.2/−11.9	8.3/19.0	90/92	−139.9/−161.6	56.0/69.5	−21.6/−45.4	69.5/101.5	74.8/87.6	31.5/31.5
Patient 6	−9.2/−14.2	99.4/75.7	84/89	−123.1/−141.6	126.1/142.1	−42.9/−24.5	35.3/45.3	22.1/17.9	34/32
Patient 7	−7.8/−3.6	17.5/6.8	74/56	−190.0/−160.2	112.5/99.4	−43.9/−33.2	67.5/53.1	57.0/48.7	32.5/32.5
Normal Reference	-	218.5 ± 148.3	85.9 ± 14.1	−210.1± 172.1	347.0 ± 134.1	−36.4 ± 25.8	109.8 ± 67.8	121.4 ± 65.5	26.3 ± 3.8

Abbreviation: IT = implicit time; NA = not available; OD = right eye; OS = left eye. * Patient 4 did not have ERG because he was too young to cooperate with the exam.

**Table 4:** Summary of the VUS variant details, minor allele frequency, and pathogenicity predictions.

	Gene	cDNA	Protein	Clinical Significance (ClinVar)	ACMG/AMP Classification	Allele Frequency (Gnomad v2.1.1)	Phylo P100 Way ^†^	SIFT	PolyPhen	CADD	REVEL	MetaLR	Mutation Assessor
Patient 2	*GRM6*	c.547G>A	p.Asp183Asn	VUS	PM2+PP3	0.00000798	7.798	Deleterious	Possibly damaging	Likely benign	Damaging	Damaging	High impact
	*GRM6*	c.575G>A	p.Arg192Gln	VUS	PM2+PP3	0.0000837	7.798	Deleterious	Damaging	Likely deleterious	Damaging	Damaging	High impact
Patient 5	*TRPM1*	c.2921T>G	p.Leu974Arg	VUS	PM2+PP3	0.0000318	9.317	Deleterious	Possibly damaging	Likely benign	Possibly damaging	Damaging	Medium impact
	*TRPM1*	c.730G>A	p.Ala244Thr	VUS	PM2	0.0000441	6.160	Tolerated	Possibly damaging	Likely benign	Likely benign	Tolerated	Low impact

^†^ Positive values indicate conservation and greater values indicate higher conservation; values greater than 7.2 indicate high conservation.

**Table 5:** Summary of clinical features compared to other cohorts.

Population Cohort	Type	Gene	Average Age	Sample Size	Visual Acuity * (Snellen)	logMAR (Mean ± SD)	Spherical Equivalent (D)	Nystagmus (%)	Strabismus (%)
Korean [19]	Complete	*TRPM1*	4	1	20/50	0.1 ± 0	−7	0	0
	Complete	*NYX*	2.5	2	20/100	0.65 ± 0	−7.8	50	50
	Incomplete	*CACNA1F*	2.86	14	20/123	0.74 ± 0.22	−2.34	71	36
British [20]	Complete	*GRM6*	23.9	9	20/64	0.51 ± 0.57	−5.375	55.56	22.22
Dutch [21]	Complete	*TRPM1*	16	2	20/51	0.41 ± 0.16	−3.75	100	n/a
	Complete	*NYX*	24.8	6	20/31	0.21 ± 0.21	−9.95	66.7	n/a
	Incomplete	*CACNA1F*	22.6	13	20/55	0.44 ± 0.29	−5.56	61.5	n/a
Slovenian [9]	Autosomal dominant	*RHO*	24.3	3	20/20	0 ± 0	n/a	0	0
Taiwanese This paper	Autosomal dominant	*RHO*	22	1	20/20	0 ± 0	−3.5	0	0
	Complete	*GRM6*	28	1	20/200	1.0 ± 0	−1.5	100	100
	Complete	*TRPM1*	9	3	20/200	0.813 ± 0.32	−5.125	100	100
	Incomplete	*CACNA1F*	26	1	20/500	1.40 ± 0	+4.125	100	100
	Complete	*NYX*	22	1	20/20	0 ± 0	−7.25	0	0

* Visual acuity and spherical equivalent are the mean values of both eyes’.
